# The *Anaplasma ovis* genome reveals a high proportion of pseudogenes

**DOI:** 10.1186/s12864-018-5374-6

**Published:** 2019-01-21

**Authors:** Zhijie Liu, Austin M. Peasley, Jifei Yang, Youquan Li, Guiquan Guan, Jianxun Luo, Hong Yin, Kelly A. Brayton

**Affiliations:** 1 State Key Laboratory of Veterinary Etiological Biology, Key Laboratory of Veterinary Parasitology of Gansu Province, Lanzhou Veterinary Research Institute, Chinese Academy of Agricultural Sciences, Lanzhou, People’s Republic of China; 2 Program in Genomics, Department of Veterinary Microbiology and Pathology, Washington State University, Pullman, WA 99164-7040 USA; 3 Jiangsu Co-innovation Center for Prevention and Control of Important Animal Infectious Diseases, Yangzhou, China

**Keywords:** Genome sequence, Comparative genomics, Vaccine development, Diagnostic assay, Rickettsial pathogen

## Abstract

**Background:**

The genus *Anaplasma* is made up of organisms characterized by small genomes that are undergoing reductive evolution. *Anaplasma ovis*, one of the seven recognized species in this genus, is an understudied pathogen of sheep and other ruminants. This tick-borne agent is thought to induce only mild clinical disease; however, small deficits may add to larger economic impacts due to the wide geographic distribution of this pathogen.

**Results:**

In this report we present the first complete genome sequence for *A. ovis* and compare the genome features with other closely related species. The 1,214,674 bp *A. ovis* genome encodes 933 protein coding sequences, the split operon arrangement for ribosomal RNA genes, and more pseudogenes than previously recognized for other *Anaplasma* species. The metabolic potential is similar to other *Anaplasma* species. *Anaplasma ovis* has a small repertoire of surface proteins and transporters. Several novel genes are identified.

**Conclusions:**

Analyses of these important features and significant gene families/genes with potential to be vaccine candidates are presented in a comparative context. The availability of this genome will significantly facilitate research for this pathogen.

## Background

*Anaplasma ovis* is a Gram-negative, tick transmitted rickettsial pathogen that causes anaplasmosis of sheep, goats and wild ruminants throughout Asia, Africa, Europe and the U. S. [1, 2]. It is typically more pathogenic in goats, and only rarely infects cattle [3–7]. *A. ovis* infects the erythrocyte where it is phenotypically similar to, but does not provide protection against *Anaplasma marginale* infection [5]. *A. ovis* is thought to only induce mild clinical disease, and thus, serious consideration of this pathogen has not been undertaken despite widespread infection in livestock [8]. Losses in productivity, though minor in the individual animal, can be compounded by the fact that infection is pervasive, and the disease state can be exacerbated by stressors such as: co-infections, heavy tick burden, elevated temperatures, vaccination, deworming, and animal movement [2, 8]. Therefore, the economic impact of this neglected pathogen may be underestimated.

*Anaplasma ovis* infection has been detected by examination of Giemsa stained blood smears, complement-enzyme linked immuno sorbent assay (cELISA) (Msp 5) and PCR (*msp4*) [8]. The former two tests, while cheap and simple, do not discriminate to species level, and often a diagnosis of *A. ovis* as the causative agent is assumed when screening ovine/caprine animals [9]. While the species-specific *msp4* PCR has been used in several studies, access to a greater array of diagnostic targets would be of benefit for epidemiological studies and researchers.

Organisms in the order Rickettsiales are small, obligate intracellular bacteria that typically have genomes from 1.2–1.5 Mb [10]. These small genomes are thought to result from reductive evolution and long intracellular association with a host [11–13]. The obligate intracellular nature of rickettsial organisms makes them difficult to culture, and to obtain host-free pathogen DNA. The genome of a number of rickettsial pathogens, including representatives of several *Anaplasma* species, have had their genomes sequenced which has catalyzed research for these pathogens [14–17]. In this study we have generated the complete genome sequence for the *A. ovis* Haibei strain using a combination of Solexa and Pacific Biosciences sequencing technologies. The genome has been annotated and manually curated and compared to *A. marginale* and *A. centrale*, it’s closest relatives [14, 16]. The genome features and information about important gene families is presented. We used structural prediction tools to infer novel insights about several hypothetical proteins.

## Results

### *General Features of the* A. ovis *Genome*

**Fig. 1:**
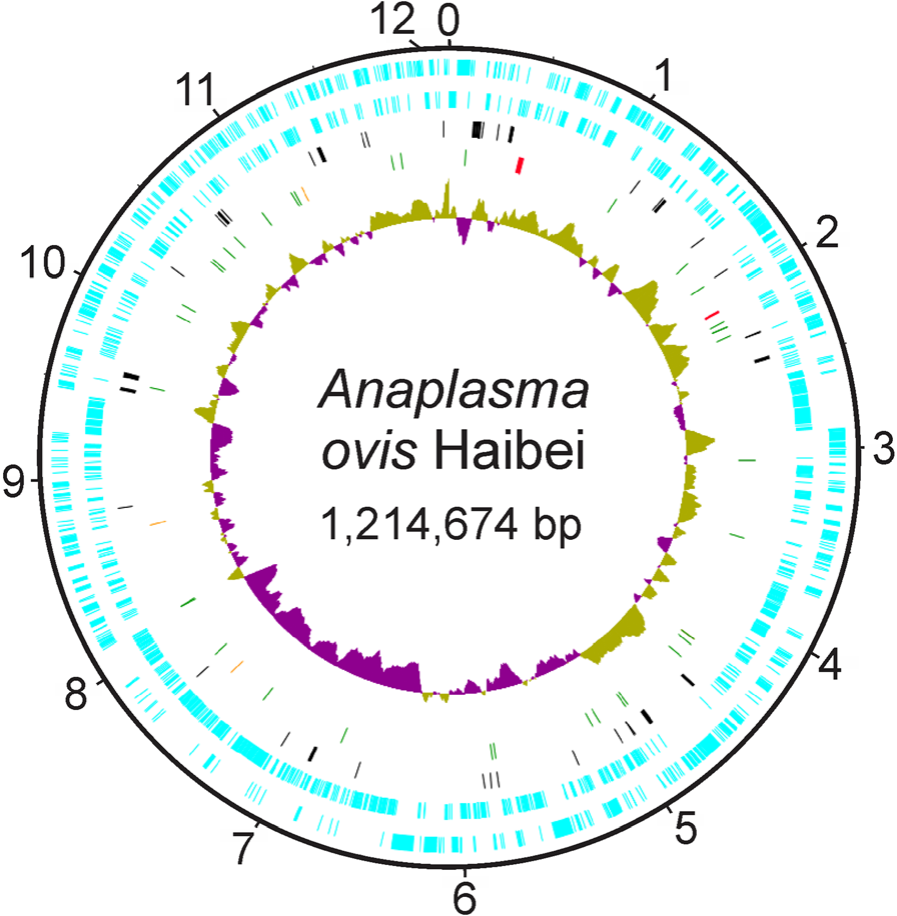
Circular map of the *Anaplasma ovis* genome. The outermost ring shows the genome size in 100 kb increments. Moving inwards, the light blue marks indicate the coding sequences on the forward and reverse strands, the black marks indicate pseudogenes, the next ring contains the noncoding RNAs with tRNAs in green, rRNAs in red and other ncRNAs in orange. The innermost ring shows the GC skew, with olive green being positive and purple being negative. Image made in DNAplotter

**Fig. 2:**
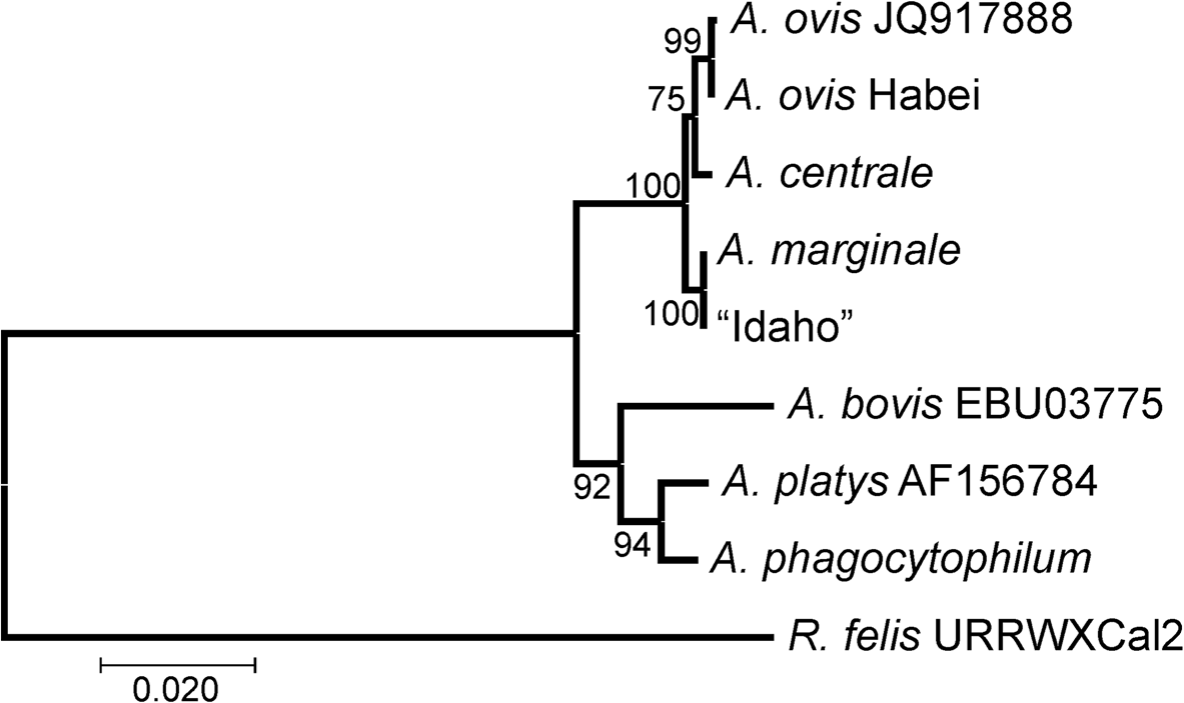
Phylogenetic tree based on 16S sequences. Accession numbers are given for 16S sequences from *A. platys* and *A. bovis*. Other 16S sequences were taken from whole genomes with accession numbers as follows: *A. centrale*: CP001759; *A. ovis* Haibei: CP015994; *A. marginale*: CP00030; *A. phagocytophilum*: CP000235. *Rickettsia felis* (CP000053) was used as an out group. The evolutionary history was inferred using the Neighbor-Joining method [61]. The optimal tree with the sum of branch length = 0.23834825 is shown. The percentage of replicate trees in which the associated taxa clustered together in the bootstrap test (1000 replicates) are shown next to the branches [62]. The evolutionary distances were computed using the Kimura 2-parameter method [63] and are in the units of the number of base substitutions per site. Evolutionary analyses were conducted in MEGA7 [64]. This tree is representative of the Maximum Likelihood tree

The manually curated final genome of *A. ovis* strain Haibei contains a circular chromosome of 1,214,674 bases and no plasmids, and has an overall G + C content of 49% (Table 1; Fig. 1). Within the seven recognized species in the genus *Anaplasma*, *A. marginale* (the type species), *A. centrale* and *A. ovis* are more closely related to each other than to the other species in the genus (Fig. 2). A G + C content of ~ 50% is typical for this cluster of related species, with *A. phagocytophilum* having a slightly lower G + C content. As with all *Anaplasma* (Rickettsiales) species reported to date, *A. ovis* contains a single split operon encoding the ribosomal RNA genes, with 23S and 5S genes being transcribed as one polycistron and the 16S gene being transcribed separately. The 37 tRNA genes represent all 20 amino acids (aa). There is a high coding density of 82% and a relatively large average gene length at just over 1 kb.

**Table 1:** General features of *Anaplasma* genomes

	*A. ovis* Str Haibei	*A. marginale* Str St. Maries	*A. centrale* Str Israel	*A. phagocytophilum* Str HZ
Total Bases	1,214,674	1,197,687	1,206,810	1,471,282
CDS Count	933	949	925	1066^a^
tRNAs	37	37	37	37
nc RNAs	3	3	3	3
rRNAs	3	3	3	3
tmRNA	1	1	1	1
Pseudogenes	44	20	24	111
Functional pseudogenes	15	14	16	75
Coding %	83.0	85.4	84.4	68.2
GC %	49.0	49.9	50.0	42.6

^a^This is using the RefSeq NC_007797.1 which has fewer CDSs annotated than the original deposition CP000235 (1264)

### Pseudogenes

Rickettsial organisms have been described as undergoing reductive evolution, a process whereby they are losing genes over time. The *R. conorii* genome was described as actively undergoing this process with 37 genes present as “split Open Reading Frames (ORFs)”, a process the authors described as the first step in the reductive evolution pathway [12]. The idea being that a gene is mutated with a single base deletion or insertion that results in a frameshift that alters the coding capacity of the gene, (ie, most likely renders the gene non-functional) and a results in smaller open reading frames, in two different frames. Over time, this non-functional gene would acquire more mutations and eventually it would be removed from the genome or changed beyond recognition. The *A. marginale* St. Maries strain genome contains four such split ORFs, and the Florida strain genome also has four split ORFs, albeit different genes [14, 18]. We ruled out sequencing errors as a contribution to the observed changes. Since these *A. marginale* genomes were completed, high throughput sequencing technologies were developed, which have a tendency to err when sequencing homopolymeric tracks, and these types of frameshift errors are more frequent [19]. When the initial auto-annotation of the *A. ovis* genome was completed, 47 genes were found to contain frameshifts. Some of these were deemed to be incorrect (the gene was simply annotated in the wrong frame), but all that appeared real were checked by PCR, and corrected, based on the resulting sequence. The final annotation contains seven genes that contain frameshifts (i. e. split ORFs; Table 2). Interestingly, one of the *A. ovis* split ORFs occurs in mutL (AOV_01085), which also was split in the *A. marginale* St. Maries strain genome (this gene was not split in other *A. marginale* genomes). The other genes containing frameshifts are AOV_00810, mtgE (AOV_02395), fadB (AOV_02655), AOV_02780, lytB (AOV_02875), and AOV_03945. In the case of genes encoding hypothetical proteins, these frameshifts are recognized as these sequences contain a frameshift as compared to *A. marginale*; however, it should be noted that when there are not many sequences in the database, these cases should be examined carefully. For example, in the case of AOV_00810, there is a potential ORF in a single reading frame that may be the “real” gene.

**Table 2:** Functional and Classical Pseudogenes in *A. ovis*

Ψ^a^				Classical Ψ		Functional Gene	
Locus ID	name	Length^b^	Functional Ψ	frameshift	truncated	Locus ID	Length
AOV_00155	msp2Ψ1	336	X			msp2 AOV_04300	1215
AOV_00600	msp2Ψ2	675	X				
AOV_03655	msp2Ψ3	750	X				
AOV_04515	msp2Ψ4	396	X				
AOV_04865	msp2Ψ5	414	X				
AOV_05215	msp2Ψ6	483	X				
AOV_04490	msp2Ψ7	579	X				
AOV_00040	msp3Ψ1	2043	X			msp3 AOV_03915	2426
AOV_00045	msp3Ψ2	1602	X				
AOV_00050	msp3Ψ3	1851	X				
AOV_00145	msp3Ψ5	2148	X				
AOV_00605	msp3Ψ6	1752	X				
AOV_03985	msp3Ψ8	2691	X				
AOV_04500	msp3Ψ9	2127	X				
AOV_04855	msp3Ψ10	1932	X				
AOV_00810	H^c^	445		X		–	
AOV_01085	mutL	1892		X		–	
AOV_02395	mgtE	1348		X		–	
AOV_02655	fadB	1131		X		–	
AOV_02780	H	1690		X		–	
AOV_02875	lytB	919		X		–	
AOV_03945	H	2421		X		–	
AOV_00065	msp3Ψ4	438			X	AOV_03915	2426
AOV_00115	omp14	573			X	AOV_00035	1158
AOV_00515	sucD	223			X	AOV_00660	885
AOV_01020	thiE	384			X	AOV_01015	1026
AOV_01025	thiE	252			X		
AOV_01820	msp1a	1611			X	AOV_01835	2175
AOV_01970	infB	387			X	AOV_02590	2502
AOV_01975	infB	702			X		
AOV_02035	pccB	2035			X	AOV_02715	1533
AOV_02120	purA	195			X	AOV_03080	1281
AOV_02180	purA	195			X		
AOV_02195	trxB2	228			X	AOV_02205	1023
AOV_02200	trxB2	669			X		
AOV_02415	pepA	1158			X	AOV_03525	1497
AOV_02440	pepA	540			X		
AOV_02835	lpdA	420			X	AOV_02845	1416
AOV_03085	ObgE	747			X	AOV_02115	1044
AOV_03660	msp3Ψ7	444			X	AOV_03915	2426
AOV_04305	virB2a	342			X	several	$\overline{x}$ =378
AOV_04495	virB2a	318			X		
AOV_04995	omp6	516			X	AOV_04980	1179
AOV_05010	H	333			X	–	

^a^Ψ = pseudogene

^b^Length is given in base pairs

^c^H = hypothetical

Other pseudogenes include 20 genes with potential defects to render them nonfunctional. Some are truncated versions of full length genes and others contain internal stop codons (Table 2). Notable among these is a pseudogene for *msp1a* (AOV_01820) which is truncated at the 5′ end and at the 3′ end. This is the first time we have observed a pseudogene for *msp1a* in any species. The pseudogene is 2.8 kb upstream from the full length *msp1a* expression site, with the gene for elongation factor 4 between the pseudogene and the functional *msp1a* gene. Amongst the other pseudogenes of this type, there was a truncated version of *omp14* (AOV_00115); a gene annotated as *omp6* (AOV_04995) which is a truncated version of *omp10* (AOV_04980), but we retained the name since it is annotated like this in the *A. marginale* genome; two truncated pseudogenes ([AOV_01970, AOV_01975], [AOV_02415, AOV_02440], [AOV_01020, AOV_01025] [AOV_02120 and AOV_02180] and [AOV_02195 and AOV_02200]) each for *infB* (AOV 02590), *pepA* (AOV_03525), *thiE* (AOV_01015) *purA* (AOV_03080) and *trxB2* (AOV_02200). There were truncated pseudogenes for methylmalonyl-CoA carboxyltransferase (AOV_02035), *sucD* (AOV_00515), GTPase ObgE (AOV_03085), and dihydrolipoyl dehydrogenase (AOV_02835). There was a pseudogene (AOV_05010) for a hypothetical protein that had internal stop codons as compared to the *A. marginale* homolog. There were two pseudogenes for the *virB2* genes, one contained internal stop codons (AOV_04305), while the other was a little shorter at the 5′ end than most virB2s. However, there are alternative start codons that could be used (AOV_04495), i. e. it is possible that this gene could encode a functional product by using an alternative start codon.

**Fig. 3:**
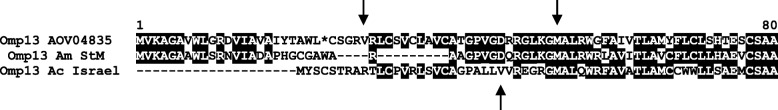
Omp13 alignment showing potential start sites. The first 80 codons of the *A. ovis* Omp13 sequence are shown on the top line (Omp13 AOV_04835), and the corresponding *A. marginale* sequence (St. Maries strain) in the middle and the *A. centrale* (Israel strain) sequence on the bottom line. The asterisk shows the position of the stop codon in the *A. ovis* sequence. The arrows show potential start sites downstream from the stop codon. In the *A. centrale* sequence, the arrow shows the annotated start codon (GTG), however the sequence upstream from this start is open and contains an in frame methionine start codon. The amino-terminal sequence is not conserved. The methionine at the right most arrow is conserved in all three species, and could be the appropriate start codon for this protein

*Omp13* was auto-annotated as a pseudogene as it contains a stop codon at base 69 relative to the putative start codon. We verified this sequence by amplifying and resequencing this gene. However, it should be noted that there are two potential start codons 84 and 156 bases downstream from the annotated start codon, both downstream from the “internal” stop, and either of these could be the actual true start for this protein. The *A. centrale omp13* sequence is shorter than the *A. marginale* and *A. ovis* sequences, and alignment of the deduced amino acid sequences for these proteins is shown in Fig. 3. We predict that the conserved Methionine highlighted in Fig. 3 is the appropriate start codon for this protein in all three species. Therefore we have not annotated this as a pseudogene.

A second type of pseudogene was referred to as a “functional pseudogene” during the annotation of the *A. marginale* genome [14]. The functional pseudogenes are truncated versions of either *msp2* or *msp3* which, in their current location, cannot express protein, but they can be recombined by gene conversion into the respective expression site to create a new variant of each gene. The St. Maries strain of *A. marginale* has seven functional pseudogenes for *msp2* and another seven for *msp3*. The number of functional pseudogenes appears to vary somewhat by strain. *Anaplasma ovis* has seven functional pseudogenes for *msp2* and eight for *msp3*. In addition, there are two classical pseudogenes for *msp3*, as these genes are so truncated that they do not contain the necessary components for recombination into the expression site (Table 2).

Altogether there are 44 pseudogenes in the *A. ovis* genome, including genes that contain frameshifts, truncated genes, genes with internal stop codons and the functional pseudogenes for *msp2* and *3*. We suspect that there are additional pseudogenes in a large family of genes containing a motif (see ‘family with motif’ section) as some of the genes are quite truncated and do not appear to be full length compared to the rest of the members of the family. However, as these are all hypothetical proteins, and we do not know their function, we cannot assess whether these truncated gene copies are likely to be functional. Still, a significant portion of the genome, over 3% of the coding capacity (> 40 kb) corresponds to pseudogenes.

### Metabolic potential

*Anaplasma ovis* has the coding capacity for gluconeogenesis but not glycolysis. The genome encodes all the necessary enzymes for the TCA cycle, fatty acid biosynthesis, de novo biosynthesis of purines and pyrimidines, and the nonoxidative pentose phosphate pathway. Many amino acid biosynthetic pathways were not complete. The metabolic reconstruction is very similar to both *A. marginale* and *A. centrale*.

### Transporters

The genome annotation finds 76 genes/proteins with a role in transport, a similar number to other *Anaplasma* species (Table 3). The sec pathway for generalized secretion of proteins is present with the exception of *secM*, a monitoring protein. The twin arginine targeting (Tat) system functions to translocate folded proteins and/or cofactor containing proteins across the membrane. Tat systems consist of two or three subunits integrated into the cytoplasmic membrane – either TatA and TatC or TatA, TatB and TatC [20]. Both *tatA* (AOV_01285) and *tatC* (AOV_02605) are present in the *A. ovis* genome. A gene for TolC is also present in the *A. ovis* genome. TolC is a multi-purpose pore-forming protein that can be used in the Type I Secretion System (T1SS). TolC is recruited to a membrane fusion protein (MFP) that crosses the inner membrane and bridges it to the outer membrane, after the MFP and an ABC transporter (in the inner membrane) have contacted a substrate [21]. Many T1SSs secrete toxins, and appear to have pairs of MFPs and ABC transporters; however, none of these systems have been identified in *A. ovis* or other closely related species, despite identification of many ABC transporters.

**Table 3:** Transporters in *Anaplasma* species

	*A. ovis* Haibei	*A. centrale* Israel	*A. marginale* St. Maries	*A. phagocytophilum HZ*
Genome Size(kb):	1234.92	1206.81	1197.69	1471.28
Total Transporter Proteins:	76	78	77	73
No. of Transporters per kb genome:	0.06	0.06	0.06	0.05
ATP-Dependent	33 (43%)	38 (49%)	35 (45%)	33 (45%)
ATP-binding Cassette (ABC) Superfamily	21	24	22	20
Bacterial Competence-related DNA Transformation Transporter (DNA-T) Family	1	2	2	1
H^+^ − or Na^+^ −translocating F-type, V-type and A-type ATPase (F-ATPase) Superfamily	6	7	7	8
Type IV (Conjugal DNA-Protein Transfer or VirB) Secretory Pathway (IVSP) Family	5	5	4	4
Ion Channels	2 (2%)	2 (3%)	2 (3%)	1 (1%)
H^+^ − or Na^+^ −translocating Bacterial Flagellar Motor /ExbBD Outer Membrane Transport Energizer (Mot-Exb) Superfamily	2	2	2	1
Secondary Transporter	34 (42%)	31 (40%)	33 (43%)	32 (44%)
Auxin Efflux Carrier (AEC) Family	1	1	0	1
Alanine or Glycine:Cation Symporter (AGCS) Family	1	1	1	1
Autoinducer-2 Exporter (AI-2E) Family (Formerly PerM Family, TC #9.B.22)	1	1	1	1
Amino Acid-Polyamine-Organocation (APC) Family	1	0	1	0
Cation Diffusion Facilitator (CDF) Family	1	1	1	1
Monovalent Cation:Proton Antiporter-2 (CPA2) Family	1	1	1	1
Monovalent Cation (K^+^ or Na^+^):Proton Antiporter-3 (CPA3) Family	10	10	10	8
Dicarboxylate/Amino Acid:Cation (Na^+^ or H^+^) Symporter (DAACS) Family	2	1	2	4
Major Facilitator Superfamily (MFS)	8	8	7	5
Multidrug/Oligosaccharidyl-lipid/Polysaccharide (MOP) Flippase Superfamily	1	1	1	1
Oligopeptide Transporter (OPT) Family	0	0	1	0
Cytochrome Oxidase Biogenesis (Oxa1) Family	0	1	1	2
Resistance-Nodulation-Cell Division (RND) Superfamily	1	1	1	1
Solute:Sodium Symporter (SSS) Family	1	1	1	1
Twin Arginine Targeting (Tat) Family	3	1	2	2
Tripartite ATP-independent Periplasmic Transporter (TRAP-T) Family	1	1	1	2
K^+^ Transporter (Trk) Family	1	1	1	1
Unclassified	7 (8%)	7 (9%)	7 (9%)	7 (10%)
HlyC/CorC (HCC) Family	1	1	1	1
Mg2^+^ Transporter-E (MgtE) Family	0^a^	1	1	1
Peroxisomal Protein Importer (PPI) Family	1	0	0	0
Tellurium Ion Resistance (TerC) Family	1	1	1	1
YggT or Fanciful K^+^ Uptake-B (FkuB; YggT) Family	4	4	4	4

^a^Gene present, but contains a frameshift

Type 4 Secretion Systems (T4SSs) are multimeric protein complexes that span the membrane and secrete proteins (and DNA) into the host cell. The genes encoding the T4SS components are called *virB1–11* and *virD4*, and have also been referred to as *rvh* to specify rickettsial *vir* genes. *Anaplasmataceae* have had an expansion of several genes that make up the complex, such that proteins that normally have a single representative for most bacteria are represented by up to 22 copies in *Anaplasma* species [22]. *Anaplasma ovis* has a T4SS that is encoded by 11 *virB2* genes, 4 *virB6* genes, 2 genes for each of *virB4*, *virB8,* and virB9, and one gene each for *virB3, virB7*, *virB10*, *virB11*and *virD4*. VirB1 and VirB5 have not been identified for any *Anaplasmaceae* [22]. The *virB2* genes come in two types and are annotated as *virB2a* and *virB2b*, with the primary difference being that the “a” type are longer at the 5′ end of the gene. The a and b type typically occur in pairs, and usually occur juxtapositioned next to an *msp2* or *3* (pseudo)gene. In addition to the 11 functional copies, there are two pseudogenes, discussed earlier. VirB6 is an integral membrane protein and has direct contact with the effector molecules as they are translocated. The expansion of *virB6* genes is thought to reflect substrate specificity [23]. Many of the T4SS genes are arranged in two operons, with one containing *virB3*, *virB4*, and *virB6–1-4*, and the other containing *virB8–1*, *virB9–1*, *virB10*, *virB11*, and *virD4*. The other genes are dispersed around the genome.

### Membrane proteins

Upon using SignalP (version 4.1; [24]) to predict signal peptides, we found that 53 proteins contained signal sequences, and analysis with TMpred [25] indicated that all of these proteins had at least one transmembrane domain. Since this number is much lower than what was predicted for *A. marginale* (163) using a previous version of the program (version 3), we reanalyzed the *A. marginale* genome with the current version, SignalP 4.1 and found only 48 sequences predicted to contain signal peptides. This analysis is in line with the relatively small number of proteins that have been predicted to be surface localized for these organisms [14, 26].

### Gene families of interest

#### The msp2 superfamily

Msp2 and 3 are surface proteins responsible for immune evasion, which they achieve by gene conversion [27, 28]. In *A. marginale*, the *msp2/3* functional pseudogenes are recombined by gene conversion into the single expression site to create immune escape variants. The *msp2* superfamily is composed of related genes that encode outer membrane proteins (OMPs) that fall into pfam01617. The *A. ovis* Haibei genome contains 17 members of this superfamily, including *omp1,4–8, 10–14*, *opag1–3*, and *msp2–4* (not counting functional pseudogenes). Missing from the genome are homologs of *omp2*, *omp3*, *omp9*, and *omp15*, which are found in *A. marginale*. Interestingly, the *A. ovis* complement of *omp* genes is similar to that of *A. centrale*. Both species are missing *omp2*, *omp3* and *omp15*. When it comes to the operon that starts with *omp10* and goes through *omp6* in *A. marginale*, the situation is a little different in each species. Recall that Omp7–9 are similar to each other, when comparing across the genes/proteins there are conserved ends and a central variable region: the amino-terminal and carboxy-terminal regions have 85–91% and 81–84% identity while the central regions have 35–51% identity. *Anaplasma centrale* contains *omp10*, and a single gene referred to as *omp7*, followed by a truncated version of *omp9*. In *A. ovis*, *omp10* is followed by *omp8* and *omp7*, and a truncated version of *omp10*, which we have called *omp6* as in the *A. marginale* genome. These proteins (*omps7–9*) are of interest as they have been studied as vaccine candidates [29, 30]. As with *A. marginale* and *A. centrale*, *msp2* is found at the 3′ end of an operon that also contains *opag1–3*. This operon arrangement is also seen in *A. phagocytophilum*, but is less conserved, with only two genes upstream of *msp2/P44* called *omp-1 N* and *omp-1X* (also called *p44Sup1*) [31]. It is assumed that with a similar number of pseudogenes for *msp2* and *msp3*, that gene conversion will be the operational force working in the *A. ovis* genome to provide variation in these genes.

In *A. marginale*, *msp3* is also expressed from the 3′ end of an operon, with two *virB2* genes at the start of the operon [14]. In the *A. ovis* Haibei genome, the full length *msp3* gene is positioned in close proximity to two *virB2* genes; however, the distances between each (160 bp and 290 bp) would suggest these are not typical of a polycistronic arrangement. The deduced amino-terminal sequence has 65% identity with the *A. marginale* Msp3 sequence.

#### The msp1 superfamily

Msp1 is a surface protein composed to two polypeptides, Msp1a and Msp1b. The *msp1α* gene of *A. marginale* has been used extensively as a surrogate measure for strain diversity [32], and recently, we and others have shown that the corresponding homolog in *A. ovis* can be used in the same manner [33, 34]. Like *A. centrale*, the Msp1a repeats of *A. ovis* are longer than those found in *A. marginale*, with the repeats that have been reported ranging in size from 33 to 47 aa in length. There are four repeats found in the *msp1a* gene in the *A. ovis* genome encoding repeats of 49, 55, 55 and 39 aa. While these appear to be longer, we suspect that the full repeat was not reported in the previous papers; when examining the repeats from one of the previous studies, we see that they can also be up to 55 aa in length (see MG642087; from [33]). Similar to the repeats in *A. centrale* Msp1aS, there are many serine and glutamine residues indicating that the repeats have a polar character. Msp1a is encoded by a single gene; however, as mentioned above, in *A. ovis*, there is a pseudogene present and care must be taken when analyzing the repeat sequences to ensure that investigators are actually analyzing the repeats from the functional copy of the gene. Downstream from the *msp1a* gene reside five copies of the *mlp* gene (*mlp1–5*) encoding the Msp1a-like protein which have four transmembrane domains that are characteristic of the carboxy-terminus of Msp1a [14].

The *A. ovis* genome contains one full length copy of *msp1b*, and a truncated copy, referred to as partial gene 1 or *msp1bpg1*. The partial gene corresponds to the 3′ ~ 70% of the full length gene. This is a similar arrangement to the *A. centrale* genome, while *A. marginale* has two complete genes and three partial genes [35].

#### The aaap family

In *A. marginale*, the *Anaplasma* appendage associated protein (Aaap) associates with actin filaments that are on the cytoplasmic face of the parasitophorous vacuole [36]. This protein is polymorphic among strains, and is characterized by repeats of “EL(K/R/D)AIDA”. The St. Maries strain genome sequence revealed two additional aaap-like proteins, or *alp1* and *alp2*, while the Florida strain had a second copy of aaap, and three *alp* genes [14, 18]. The *A. centrale* genome only had three *alp* genes, and no gene corresponding to *aaap*. The *A. ovis* genome has one *aaap* and one *alp* gene. We recently developed an indirect ELISA assay using rAaap for diagnosing *A. ovis* infection in sheep and goats [37].

Another gene/protein that drew our attention was AOV_02945, which had 14 repeats of 11 aa containing the “ELRAIDA” motif found in Aaap. The protein “hits” with Alp1 of *A. marginale* with an e score of 1e-23, but all other BLAST hits are below the threshold of significance. AOV_02945 had a conserved domain match to the Neuromodulin_N superfamily [38, 39], a family found in multiple malaria adhesins and malaria erythrocyte binding proteins.

#### AnkA

Ankyrin (Ank) repeats are 33 aa structural motifs that mediate protein-protein interactions, and are more common in eukaryotic proteins that in prokaryotic proteins. It has been observed that Ank repeat containing proteins are often effectors of the T4SS [40]. AnkA (2134 aa), an Ank repeat containing protein, in *A. ovis* is much longer than the homologs in *A. centrale* (1424 aa), *A. marginale* (1387 aa) or *A. phagocytophilum* (1232 aa). *Anaplasma ovis* AnkA contains 13 Ank domains, most similarly arranged to the domains found in *A. centrale* AnkA. The additional ~ 700 aa contains motifs identified in Conserved Domains for both the DnaJ and RNase_E_G superfamilies, which were not found in the shorter copies of AnkA in the other species; however, these hits had relatively low e values (~e-03-e-04). AnkA in *A. phagocytophilum* is one of the few known effectors of the type IV secretion system, and it translocates to the host cell nucleus where it binds DNA and nuclear proteins. As the other *Anaplasma* species infect enucleated cells in the mammalian host, it is not clear what role AnkA might play in this setting.

### Comparative genomics

**Fig. 4:**
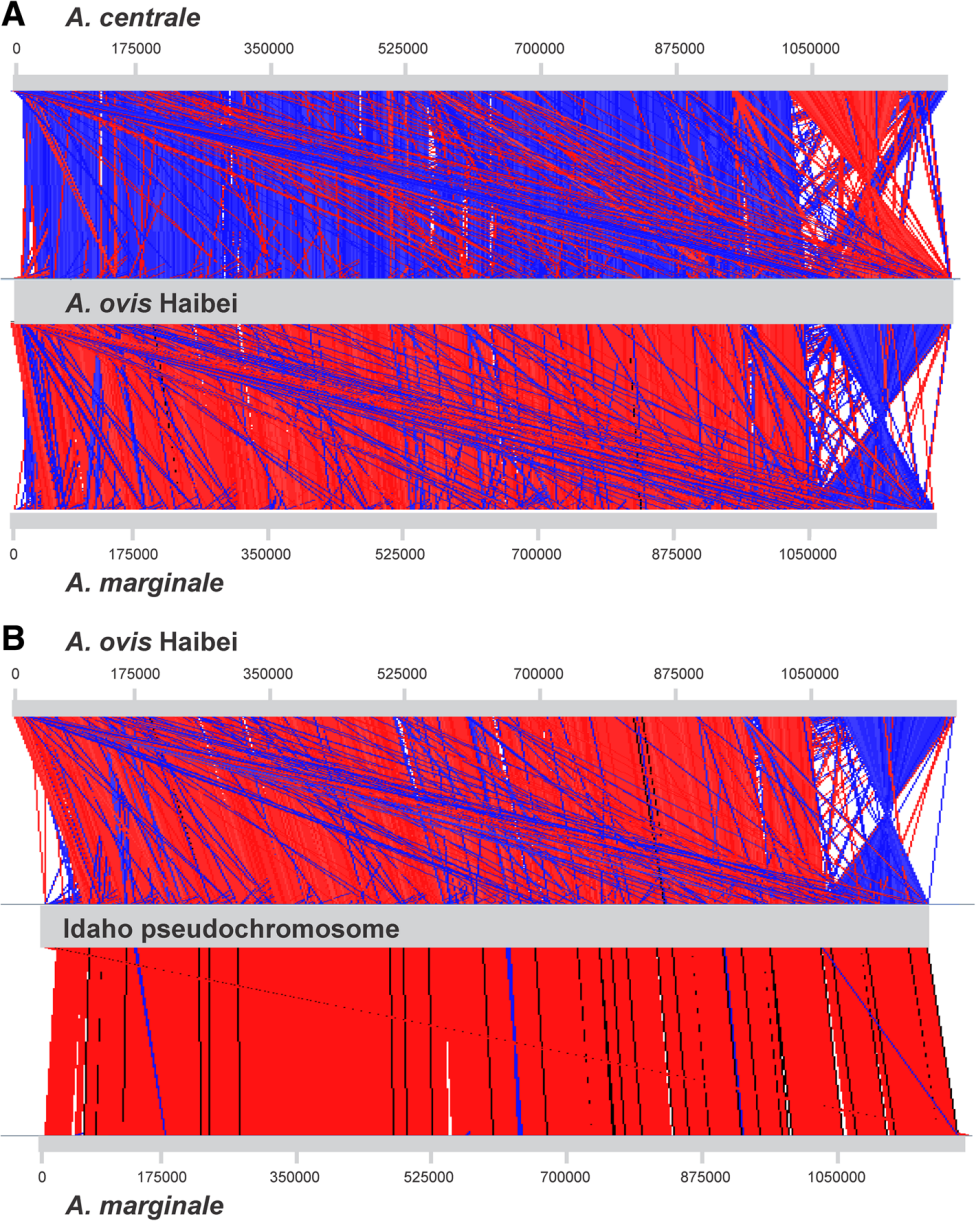
Whole Genome Alignment of *Anaplasma* species. Whole genome alignments were done using Artemis Comparison Tool. Panel **a** shows the comparison of *A. centrale* (CP001759) and *A. marginale* (CP000030) with *A. ovis* Haibei (CP015994). The *A. centrale* genome was flipped for the alignment. Panel **b** shows the alignment of *A. ovis* Haibei and *A. marginale* with the Idaho pseudochromosome (PKOE00000000). Red indicates regions of identity in the same orientation while blue indicates regions of identity with the opposite orientation

Alignment of the *A. ovis* Haibei genome with *A. centrale* and *A. marginale* shows a high degree of synteny between the genomes, although there is a large inversion of ~ 185 kb that spans the putative origin (Fig. 4a). While these genomes are highly conserved, there are a few genes/proteins present in *A. ovis* that do not appear to have homologs in *A. marginale* or *A. centrale*; these are highlighted below.

#### AOV_01200

The AOV_01200 gene is unique among *Anaplasma* species genomes. The gene is large, at 4866 bp encoding a protein of 1621 aa. The corresponding protein sequence contains 15 copies of a 46 aa repeat and another 12 copies of a shorter version of the repeat. The deduced aa sequence has highest identity (~ 37%) to Ice nucleation proteins from *Xanthomonas translucens* and *Pseudomonas syringae*. Interestingly, these are both plant pathogens. When we ran structural predictions using I-TASSER, there was a strong match to the RsaA S-layer protein from *Caulobacter crescentus* [41]. While there was a strong structural match, there was only 13.5% identity at the sequence level. Interestingly, both the ice nucleation proteins and the RsaA S-layer protein form hexamer complexes, and the matches with these proteins may reflect the conserved regions that are integral in the hexamer complex formation rather than the actual function of the protein [41–43].

#### AOV_01195

AOV_01195 encodes a protein of 487 amino acids, with best hits to AM366 in *A. marginale* or ACIS_00940 in *A. centrale*, although these genes encode proteins with > 2800 amino acids. The match is ~ 48% identity for a span of 175 amino acids. All of these proteins have no known function.

#### Family with motif

There is a family of genes/proteins that are found in four clusters throughout the genome that contain a peptide motif corresponding to the sequence “ISAVAAVAYLAVTGVSIRDLYRSCKQVIQVKEEGLVTVQSLQPVLTPITPIAGKINYGKIASA”. This motif typically occurs near the amino terminus, and the longer genes/proteins have low similarity at their carboxy-termini. The members of the family vary tremendously in size, with a few of the members of the family being quite short and which do not contain the motif, but match to other parts of deduced sequences within the family. It appears that some of these genes may be degraded forms of the longer genes; however, since we know nothing about the functionality of these sequences, we have maintained them as CDSs rather than marking them as pseudogenes. The clusters are as follows: 1) AOV_01045 (512 aa) and AOV_01050 (782 aa); 2) AOV_02420 (71 aa), AOV_02425 (118), AOV_02430 (334 aa), AOV_02450 (510 aa), with each of the smaller genes matching to different regions of AOV_02450, although they are not exact matches, and together do not correspond to a full length AOV_02450. Only AOV_02425 and AOV_02450 contain the motif. 3) AOV_02730 (437 aa), AOV_02735 (298 aa), AOV_02740 (356 aa), AOV_02745 (308 aa), AOV_02750 (80 aa), AOV_02755 (472 aa), AOV_02760 (318 aa), with only AOV_02750 missing the motif, but matching the carboxy-terminus of AOV_02755. 4) AOV_03535 (83 aa), AOV_03540 (277 aa), AOV_03545 (341 aa), AOV_03550 (475 aa), with AOV_03535 missing the motif, but matching the carboxy-terminus of AOV_02430. In pairwise comparisons, the proteins range from 0 to 89% identity with the highest match between any two proteins ranging from 62 to 89%. Altogether there are 17 genes/proteins in this family, which contrasts with *A. marginale* which has just four members of the family, AM673, AM676, AM773, and AM959. Interestingly, *A. centrale* has 10 members of the family (ACIS_00311, ACIS_00381, ACIS_00561, ACIS_00637, ACIS_00674, ACIS_00675, ACIS_00677, ACIS_00679, ACIS_00684, and ACIS_00977), however; the protein motif is shorter in this species.

#### The Idaho sequence

Recently, a draft genome sequence (accession number PKOE00000000) was deposited in GenBank reported to be the *A. ovis* Idaho strain [44]. The history of this strain is that it was isolated from sheep in 1988 by feeding *Dermacentor andersoni* ticks on naturally infected sheep and transmitting to naïve, splenectomized sheep, thus demonstrating tick transmittability [45]. The strain was isolated prior to the development of molecular tests, and (we believe) there was an assumption that if it came from a sheep, it was *A. ovis*. The cELISA based on Msp5 will not discriminate between *Anaplasma* species [45, 46]. However, with the development of molecular tests, there is a clear discrimination between *A. ovis* and *A. marginale* using genes such as *msp4*, and in fact, in 2002, when the Idaho isolate was first put into tick cell culture it exhibited an *A. ovis* Msp4 sequence indistinguishable from other published *A. ovis* Msp4 sequences [47, 48]. The Idaho draft sequence is in 43 contigs, which we BLASTed against *A. marginale* and our *A. ovis* Haibei sequences finding 99% and ~ 88% sequence identity, respectively. We bioinformatically “stitched” most of the Idaho contigs together and created a pseudochromosome (small contigs of ~ 1 kb each were left out), which was used for alignment with both an *A. marginale* genome and the *A. ovis* Haibei genome (Fig. 4b). Upon closer inspection, the Idaho genome contains an *msp1α* gene indistinguishable from *A. marginale* Virginia strain *msp1α*, encoding two repeat sequences: AB. We and other researchers have found that the form of the repeats varies between these species (discussed above), with *A. ovis* having much longer repeats similar to *A. centrale*. Further, the Msp4 sequence of the Idaho genome has 96–97% aa identity with multiple Msp4 sequences from *A. ovis*, and 100% identity with Msp4 sequences from *A. marginale* (data not shown). The 16S rRNA gene from this genome segregates with *A. marginale* upon phylogenetic analysis (Fig. 2). Finally, in the same paper reporting the Idaho genome an *A. marginale* strain Oklahoma-2 genome is also reported (accession number PKOF00000000). Strain Oklahoma-2 (Wetumka) has an *msp1α* genotype of KCH [49], however the genome sequence also contains an *msp1α* gene that has a repeat structure of AB. The Oklahoma-2 genome is in 44 contigs of surprisingly similar size to the Idaho contigs. For example, the three largest contigs from the Idaho genome are 305,977, 85,373 and 71,461 bp, while for Oklahoma-2 they are 306,104, 85,373 and 69,474 bp. There are 3 bp mismatches between the two contig 1 s from each sequence, 1 mismatch between the contig 2 s and 0 mismatches between the contig 3 s. This level of sequence identity is unprecedented for two sequences of *A. marginale* that are different strains, let alone two sequences that are different species [18, 50]. It appears that the same genome was sequenced twice. Further, with the Msp1a, Msp4 and 16S gene/protein data presented above, the most parsimonious explanation is that the “Idaho” sequence is actually an *A. marginale* sequence similar to the Virginia strain.

## Discussion

The availability of a genome sequence for *A. ovis* will facilitate the development of better diagnostic tests and a vaccine for this pathogen. When compared to other *Anaplasma* species, *A. ovis* displays marked similarities to both *A. marginale* and *A. centrale*, with some genes/regions being more similar to one species and other genes/regions being more similar to the other. An overall theme of conserved metabolic pathways and conserved synteny was obvious, with the exception of the large 185 kb inversion. Both *A. ovis* and *A. centrale* cause mild disease in their respective hosts as compared to *A. marginale*, which can be much more virulent. This difference in pathogenicity is a subtle genomic difference that has yet to be elucidated. There are no genes in *A. marginale* that are absent in these other two agents that provide overt clues to the differences in virulence. More pseudogenes were detected in the *A. ovis* genome as compared to *A. marginale* and *A. centrale*, but this is not surprising, as when the first *A. marginale* genome was sequenced there were not many close relatives to compare with, and the comparison of *A. ovis* to the other sequenced *Anaplasma* spp. helps to highlight the pseudogenes. However, even with this caveat, there appears to be more fragmented genes in *A. ovis* than in *A. marginale* and *A. centrale*. Despite growth in the databases since the first *Anaplasma* genome was completed, a large fraction (26%) of genes/proteins were still annotated as “hypotheticals”, or proteins of unknown function with no known homologs. The use of tertiary structural mapping was used to identify novel insights for a protein of unknown function that is unique among the *Anaplasmataceae*. The genome sequence has already been used to develop a novel ELISA and to design *msp1a* PCR assays for strain differentiation, and we expect that further advances will be enabled by having this genome available.

## Conclusions

*Anaplasma ovis* is an understudied ricketsial pathogen of ruminants that is closely related to *A. marginale* and *A. centrale*. This is the first complete *A. ovis* genome sequence which demonstrates a high degree of synteny with closely related *Anaplasma* species. Indeed, many features of the genome are conserved with these close relatives, such as genome size (1.2 Mb), the split operon arrangement for ribosomal RNA genes, metabolic potential and small repertoire of surface proteins and transporters. What stands out is the larger number of pseudogenes encoded in this genome as compared to it’s close relatives and several novel genes not seen in other Anaplasmataceae.

## Methods

### *Origin of* Anaplasma ovis *Haibei isolate*

*Anaplasma ovis* strain Haibei was first detected and identified by light microscopy examination of thin blood smears from a dying sheep in Haibei County in Qinghai Province. In addition, amplification with MSP45/MSP43 primers was performed according to [51]. Five ml of blood from the infected sheep was collected into a sterile EDTA-K_2_ anticoagulant tube and inoculated into a splenectomized sheep via the jugular vein as soon as the blood arrived at the laboratory. Blood from the experimental sheep was examined daily by light microscopy. When the bacteremia reached 15%, blood was collected and 5 ml aliquots, supplemented to 8% dimethyl sulfoxide (DMSO) and cryopreserved in liquid nitrogen.

### *Propagation of* a. ovis

Two three-month-old sheep were purchased from Chengye farming cooperative in Jingtai County, Gansu Province. The sheep were screened for the absence of *A. ovis*, *Babesia* and *Theileria* for a month before conducting animal experiments by weekly examination of blood smear using light microscopy and previously described PCR protocols specific for each pathogen [51–53]. Sheep No. 007 was held as a backup in case the necessary samples were not obtained from sheep No. 008. Sheep No. 008 was splenectomized to ensure rapid initiation and propagation of the infection. Infection was by intravenous inoculation of 10 ml of the cryopreserved, *A. ovis*-infected blood (approximately 15% bacteremia). When the bacteremia reached approximately 15%, venous blood from the sheep was harvested in a sterile flask containing anticoagulant (EDTA). Sheep No. 008 died from infection 3 days after a blood sample was collected for the project.

### Bacterial purification

Bacterial purification was as previously described [37]. Red blood cells (RBCs) were separated by centrifugation at 1000×g for 10 min, and the upper layer containing the white blood cells (WBCs) was discarded. The packed RBCs were suspended in phosphate-buffered saline (PBS, pH 7.2), and the remaining WBCs were removed using a commercial leucocyte filter (Nanjing Shuangwei Biotechnology, Nanjing, China). The flow-through was centrifuged as above, and the supernatant was discarded. The harvested RBCs were suspended in four volumes of PBS containing 7% glycerin and placed at room temperature for 30 min, and then centrifuged again to harvest the RBCs. The cells were then added to a flask containing four volumes of physiological saline to let the cells lyse completely. The lysate was centrifuged at 1000×g for 10 min to remove cell debris. The supernatant was then centrifuged at 10,000×g for 30 min to pellet the bacteria. The pellet was washed three times with physiological saline by centrifugation at 10,000×g for 10 min. The resulting pellet was used for DNA preparation.

### DNA preparation

DNA was extracted using a genomic DNA extraction kit (Qiagen, Hilden, Germany) according to the manufacturer’s instructions resulting in 1 ml of sample in Elution buffer. The DNA concentration was 296 ng/μl with an OD260/280 ratio of 1.92 using a Quant-iTTM dsDNA HS Assay Kit (Thermo Fisher Scientific, Beijing China).

### Genome sequencing

A 20-kb genomic DNA library was prepared suitable for P6/C4 chemistry using the SMRT bell template preparation kit 1.0 according to the manufacturer’s protocol. The *A. ovis* Haibei genome was sequenced using the PacBio single-molecule real-time (SMRT) sequencing technology using one SMRT cell on the PacBio RSII sequencing platform (Pacific Biosciences, Menlo Park, CA, USA; BGI-Shenzhen, Shenzhen, China). A total of 76,050 reads with a mean read length of 8372 bp were obtained. To minimalize the single-pass error generated by the PacBio sequencing, three DNA libraries were constructed with the insert sizes of 500 bp, 2000 bp, or 6000 bp, and were sequenced according to the standard protocols for the Illumina Hiseq 4000 platform (BGI-Shenzhen, Shenzhen, China). A hybrid approach was used to assemble and finish the genome using hierarchical genome-assembly process (HGAP) and automated workflows [54].

### Annotation

The assembled genome was submitted for annotation to the NCBI Prokaryotic Genome Annotation Pipeline (PGAP) [55]. This pipeline uses a series of programs to call genes, predict proteins, assign functional annotation, identify frameshifts, and non-coding RNAs. This annotation was manually curated, several frameshifts that were detected on the first pass were checked and corrected, where necessary. The metabolic potential was assessed using the Kyoto Encyclopedia of Genes and Genomes (KEGG) [56]. The transporters were analyzed using TransportDB 2.0 [57]. Repeats were assessed using Rapid Automatic Detection and Alignment of Repeats (RADAR) in protein sequences [58]. Protein structures were modeled with I-TASSER [59]. Whole genome alignments were done using Artemis Comparison Tool [60]. MEGA7.0 was used to generate phylogenetic trees. Functional pseudogenes are truncated copies of *msp2* or *msp3* and are annotated from stop codon to stop codon. Classical pseudogenes were detected either due to a frameshift (addition or deletion of a single base pair) within an otherwise complete gene, or when the PGAP annotated a truncated gene as an incomplete version of a known, full length gene.

The genome sequence has been deposited in GenBank with Accession number CP015994.

## Abbreviations

Ank: Ankyrin repeat domain
DMSO: Dimethyl sulfoxide
HGAP: Hierarchical genome-assembly process
KEGG: Kyoto Encyclopedia of Genes and Genomes
MFP: Membrane fusion protein
Msp: Major surface protein
OMP: Outer membrane protein
ORF: Open reading Frame
PBS: Phosphate-buffered saline
RADAR: Rapid Automatic Detection and Alignment of Repeats
RBC: Red blood cell
T1SS: Type 1 secretion system
T4SS: Type 4 Secretion System
WBC: White blood cell
